# Understanding the Mysterious M2 Macrophage through Activation Markers and Effector Mechanisms

**DOI:** 10.1155/2015/816460

**Published:** 2015-05-18

**Authors:** Tamás Rőszer

**Affiliations:** Institute for Comparative Molecular Endocrinology, Center of Biomedical Research, University of Ulm, Helmholtzstrasse 8/1, 89081 Ulm, Germany

## Abstract

The alternatively activated or M2 macrophages are immune cells with high phenotypic heterogeneity and are governing functions at the interface of immunity, tissue homeostasis, metabolism, and endocrine signaling. Today the M2 macrophages are identified based on the expression pattern of a set of M2 markers. These markers are transmembrane glycoproteins, scavenger receptors, enzymes, growth factors, hormones, cytokines, and cytokine receptors with diverse and often yet unexplored functions. This review discusses whether these M2 markers can be reliably used to identify M2 macrophages and define their functional subdivisions. Also, it provides an update on the novel signals of the tissue environment and the neuroendocrine system which shape the M2 activation. The possible evolutionary roots of the M2 macrophage functions are also discussed.

## 1. Introduction

Macrophages are frontier soldiers of innate immunity and are also indispensable players in organ development, tissue turnover, and regeneration [1–3]. Due to their immune surveillance role, macrophages sense a wide spectrum of stimuli, spanning from viral, microbial and parasite antigens, immune complexes, and apoptotic or necrotic cells to various mediators released by other cells [4–7]. In response to the stimulus they sense, macrophages are being activated, which allows them to combat the pathogens, exert an immunomodulatory role, and maintain tissue integrity [4, 8, 9]. In the recent decade a model has been developed which describes the complex mechanism of macrophage activation as a polarization towards two opposite states, the M1 or classical, and the M2 or alternative activation [4, 8].

The M1 activation is induced by intracellular pathogens, bacterial cell wall components, lipoproteins, and cytokines such as interferon gamma (IFN-*γ*) and tumor necrosis factor alpha (TNF-*α*). The M1 macrophages are characterized with inflammatory cytokine secretion and production of nitric oxide (NO), resulting in an effective pathogen killing mechanism [4, 8, 10, 11]. The M2 activation is induced by fungal cells, parasites, immune complexes, complements, apoptotic cells, macrophage colony stimulating factor (MCSF), interleukin-4 (IL-4), IL-13, IL-10, tumor growth factor beta (TGF-*β*) [10], and various other signals being reviewed in this paper. The M2 macrophages have high phagocytosis capacity, producing extracellular matrix (ECM) components, angiogenic and chemotactic factors, and IL-10 [12, 13]. In addition to the pathogen defense, M2 macrophages clear apoptotic cells, can mitigate inflammatory response, and promote wound healing [8, 14]. They are widely termed in the current literature as anti-inflammatory, proresolving, wound healing, tissue repair, and trophic or regulatory macrophages and considered as benign opposites of the M1 activated macrophages [2, 15]. However, M2 macrophages can cause allergic inflammation, aid the growth of tumor tissues, and can be cellular reservoirs of various pathogens [8]. Also, M2 macrophages have complex roles outside the context of inflammation, such as organ morphogenesis, tissue turnover, and endocrine signaling [2, 4, 6, 16–18].

These M2 macrophage tasks have biomedical impact. For instance, sustaining the M2-like state of some tissue resident macrophages, such as Kupffer cells and adipose tissue macrophages, would diminish the production of inflammatory mediators and thus may be a therapeutic approach to treat metabolic diseases [3, 17]. Angiogenic and tissue remodeling activities of the M2 macrophages have potential use in regenerative medicine [19]. In tumor tissue, however, the abrogation of the angiogenic features of the macrophages would impede tumor growth and increase tumoricidal activities of the macrophages [20]. These examples highlight why the M2 macrophages gain the attention of immunologists, cancer biologists, and researchers of the metabolic and the evolutionary biology fields today. However, the diversity of the approaches and the interpretation of the studies make it difficult to reach a consensus on the definition of the M2 macrophage entity. The latest findings also question whether macrophage activation is a dichotomic process* in vivo*, resulting in clearly defined M1 and M2 macrophages. This makes the overview of the repertoire of the currently used M2 markers timely.

This review will discuss whether the M2 markers can be reliably used to identify M2 macrophages and define their functional subdivisions. Several novel signals have been identified which induce or impede M2 activation, and these findings are also reviewed herein. The possible evolutionary roots of the M2 macrophage functions are also discussed. The review gives emphasis to the idea that the M2 macrophage terminology covers “non-M1” macrophages, which adopt heterogeneous activation states and play a wide range of roles in immunity and tissue homeostasis.

## 2. How to Define the M2 Macrophages?

Today the M2 macrophages are identified based on the gene transcription or protein expression of a set of M2 markers. These markers include transmembrane glycoproteins, scavenger receptors, enzymes, growth factors, hormones, cytokines, and cytokine receptors with diverse and often yet unexplored functions. The majority of these markers were defined by early studies of the M2 activation, based on the observation that their gene transcription was amplified by IL-4/IL-13 and fungal or parasite infections (collectively, in conditions associated with Th2 immune response [10]). However, the correlation between the expression of the M2 markers and the functional state of the macrophages is not as strict as in the case of lineage markers of other immune cells. Also, IL-4 and IL-13 can elicit the transcription of many M2 markers in other myeloid cells also, such as in dendritic cells, mast cells, and myeloid derived suppressor cells [21–24]. This shows that the M2 markers and their IL-4/IL-13 induced expression are not exclusive hallmarks of the M2 macrophages.

**Figure 1:**
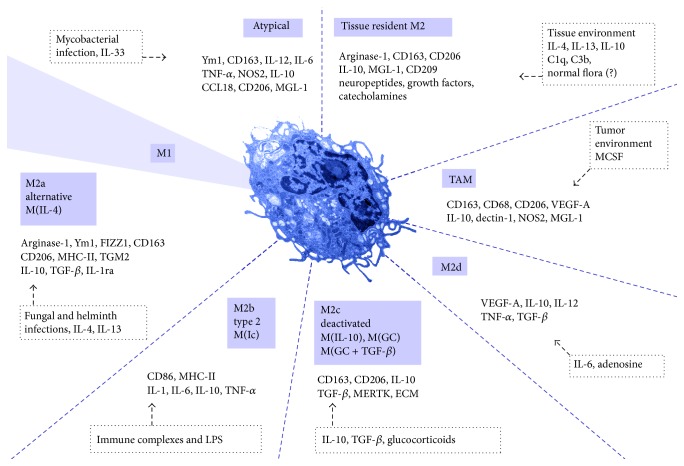
Overview of the macrophage activation states. Macrophage activation is widely considered as a polarization towards M1 or M2 states. However, the M2 activation state involves heterogeneous and functionally distinct macrophages. The diagram represents the most prevalent examples of the M2 activation and lists the markers associated with the distinct activation phenotypes. The upstream signals are labeled in dotted frames. Abbreviations are defined in the text.

Apart from IL-4/IL-13, several other stimuli and signal pathways have been recognized as inducers of M2 activation. Based on the applied stimuli and the achieved transcriptional changes, the M2 macrophages have been classified into subdivisions [4, 10]. These are M2a, M2b, and M2c subdivisions (Figure 1). Some authors distinguish the M2d macrophage type also [5, 14]. The M2a activation is a response to IL-4 and IL-13, the M2b to immune complexes and bacterial lipopolysaccharide (LPS), and the M2c to glucocorticoids and TGF-*β*. The M2d activation is a response to IL-6 and adenosines [25, 26]. There is an additional terminology to define M2 macrophages used by some authors. The M2a subtype is defined as alternative activated macrophages, the M2b as type 2 macrophages, and the M2c as deactivated macrophages (Figure 1) [10]. The deactivated terminology refers to the* in vitro* ability of macrophages to adopt M2 activation following M1 activation, thus deactivating the M1-like gene transcription [27]. The M1 macrophages—at least in murine models—have a NO burst and lack the cellular mechanisms which would allow them to survive the cytotoxic effects of NO, which questions whether such deactivation can occur* in vivo* [28, 29]. Most recently, tagging the macrophage subdivisions by defining the applied activation stimuli [10] has been proposed. In the case of the M2 macrophages these are IL-4, immune complexes (Ic), IL-10, glucocorticoids and TGF-*β* (GC + TGF-*β*), or glucocorticoids (GC). Within this classification the M2a group is termed as M(IL-4) and M2b as M(Ic), and the M2c is divided into M(IL-10), M(GC + TGF-*β*), and M(GC). This classification still fails to cover the wide range of other signals with the ability to induce M2 macrophage activation (these signals are detailed in Section 5 of this paper).

Also, the* in vivo* translation of these M2 subdivisions is difficult [8]. Tissues may contain mixed macrophage populations with a spectrum of activation states [10]. Moreover, there are atypical macrophages involved in immune response, which display both M1 and M2 associated gene transcription patterns [30, 31] or do not match the prevailing M1/M2 model [5]. Tissue resident macrophages (as detailed in Sections 3 and 4 of this paper) and tumor-associated macrophages also express M2 markers [20]. For this reason, M2 macrophages are sometimes referred to as protumorigenic macrophages, despite not being an accurate description of the wide spectrum of the M2 macrophage functions. Another limitation of the M2 activation model is that there are traits of macrophages, which do not fit into the current classification of the M2 macrophages, such as their antiviral activities, the synthesis of neurotransmitters and hormones, and lipid mediators (detailed in Section 3 of this paper).

**Figure 2:**
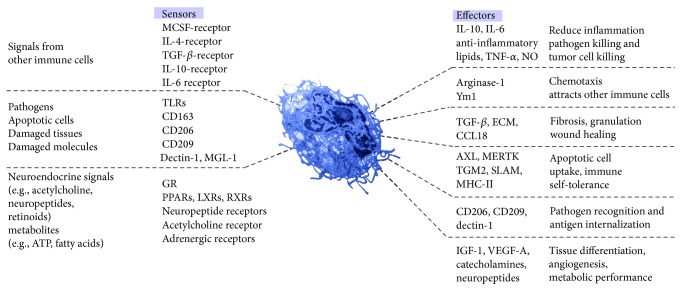
Alternative model depicting sensor and effector functions of the M2 macrophages. The macrophage phenotype is principally determined by eliciting signals, derived from immune cells, pathogens, apoptotic or damaged cells, and a wide range of chemical mediators. They act through receptors and signal pathways which elicit a wide range of effector functions. Abbreviations are defined in the text.

The current classification of the M2 macrophages gives emphasis to the activation stimuli, rather than the macrophage functions elicited by the stimuli. In the next paragraphs, I overview the effector functions of the most frequently used M2 markers (Figure 2). I also complete the list with some molecules, which are not assigned as M2 markers yet; nevertheless, their functions are related to the M2 activation.

## 3. Overview of the M2 Macrophage Markers

### 3.1. Arginase-1

Since the initial discovery of M2 macrophage activation, arginase-1 (EC 3.5.3.1), is considered as a prototypic M2 marker in the mouse [32]. Arginase-1 functions are mainly studied in the context of helminth infection and murine airway inflammation models [32]. At an evolutionary scale of the development of macrophage-like immune cells, arginase-1 may have been primarily a wound healing protein, transcriptionally induced by proteins of the TGF-*β* family [33]. In the mouse the arginase-1 encoding gene contains response elements to the IL-4 induced transcription factor signal transducer and activator of transcription-6 (STAT-6) upstream of its promoter region and its transcription is amplified by IL-4, IL-13, and TGF-*β* [33–35]. Although tissue resident macrophages constitutively express arginase-1 [33, 36], in a noninflammatory context its functions are largely unexplored. In microglia, the endogenously produced TGF-*β* sustains arginase-1 expression [37]. Moreover, signals of the tissue environment, such as netrin [38], adenosine [39], neuropeptides [40, 41], and the presence of mesenchymal cells [42] or adipocytes [43] amplify arginase-1 expression in macrophages.

Arginase-1 is an enzyme of the urea cycle, which uses the amino acid L-arginine as a substrate and produces L-ornithine and urea. Initial studies on the function of macrophage arginase-1 have emphasized that L-ornithine may enter polyamine and collagen biosynthesis, eventually promoting fibrosis and tissue healing [35]. Later it was shown that the consumption of L-arginine by arginase-1 could inhibit L-arginine dependent immune functions [44]. For instance, L-arginine depletion suppresses T-cell proliferation [35]. This may allow arginase-1 expressing M2 macrophages to dampen the CD4^+^ T cell effector response. This reduces tissue damage in the course of host defense in helminth infections [32]; however, it may worsen immunodeficiency [45]. In the cardiovascular system, there is a substrate competition of arginase-1 and NO synthase (NOS, EC 1.14.13.39) [46]. It is a widely accepted model that, in a similar manner, macrophage arginase-1 diminishes NO synthesis by consuming L-arginine, the substrate of NOS [47]. Since murine M1 macrophages produce high levels of NO to kill pathogens, it has fueled the idea that competition of arginase-1 and NOS for L-arginine would balance the macrophages between M2 and M1 activation states [48]. However, it is not likely that a rise in L-arginine availability alone would be able to shift macrophages to M1 activation. First, the NO burst of the M1 macrophages is due to the increased expression of the NOS2 (also called inducible NOS) isoform, rather than to the rise in the NO synthesis of constitutively expressed NOS isoforms [28]. The constitutively expressed NOS isoforms produce a magnitude of lower amount of NO than NOS2; thus, any sudden increase in the L-arginine pool of the macrophages would be insufficient to cause a pathogen killing NO burst [28, 49]. The distinct subcellular localization of arginase-1 and NOS2 also challenges that arginase-1 and NOS2 share the same L-arginine pool [28]. It is more feasible that the macrophage activating stimuli are the major determinants of the arginase-1 and NOS2 expression [50, 51], not allowing the substrate competition to be the active player in the determination of M2 activation.

Altogether, the possible involvement of arginase-1 in tissue healing, reduction of T-cell response, and NO levels has led to the view that it is a wound healing and anti-inflammatory enzyme in macrophages. However arginase-1 expression is amplified in inflammatory settings [52, 53], and arginase inhibition improves wound healing in the mouse [54]. For instance, arginase-1 is the most significantly upregulated gene in the mouse spinal cord during autoimmune encephalomyelitis [52]. The microglia is a major cell type expressing arginase-1 in this disease model, and inhibiting arginase-1 diminishes disease severity [52]. Similar findings have been reported in rat models of autoimmune encephalomyelitis [36]. It has also been suggested that polyamines produced by macrophage arginase-1 may attract and activate mast cells, thus promoting airway inflammation [55]. Microglia expression of arginase-1 is increased also in Alzheimer disease [56] and retinal inflammation [53]. These findings argue against the canonical anti-inflammatory attribute of arginase-1 expressing macrophages.

### 3.2. Chitinase-3-Like Protein 3 or Ym1

The chitinase-3-like protein 3 (Chi3l3), also known as Ym1, is a lectin with affinity to glycosaminoglycans such as heparin and heparan sulfate [57]. It belongs to the protein family of acidic mammalian chitinases [57, 58]. Ym1 binds chitin; however, it lacks chitinase activity and has weak beta-N-acetylglucosaminidase (EC 3.2.2.11) activity [59]. Ym1 expressing macrophage types are alveolar macrophages, splenic macrophages, bone marrow macrophages, and microglia in the mouse [60]. The Ym1-immunoreactive protein is associated with the rough endoplasmic reticulum and with needle-shaped crystalline bodies in the cytoplasm [57, 59, 61]. Macrophages synthesize Ym1 during parasitic or fungal infection [62, 63], allergy [62], eosinophilic meningitis, and meningoencephalitis [60]. In mouse peritoneal macrophages, it is induced by parasites, without steady-state expression [23, 64]. Humans lack Ym1, and its closest homolog is the eosinophil chemotactic cytokine by sequence identity; however, it is not upregulated by IL-4 [65]. Other chitinase-like proteins may have a role in human immunity; however, their assignment to macrophages is to be explored [66].

Ym1 is considered an M2 marker in the mouse [65], since IL-4 and IL-13 upregulate its expression in an IL-4 receptor and STAT-6 dependent manner [23, 24]. Its steady-state expression by the microglia is sustained by TGF-*β*, and the disruption of the microglial TGF-*β* signaling abolishes Ym-1 expression along with the upregulation of the transcription of inflammatory mediators [37]. Moreover, IFN-*γ* antagonizes the effect of IL-4 on Ym1 expression, and the lack of IFN-*γ* receptor increases Ym1 content in macrophages [67] leading to the concept that its expression is an anti-inflammatory trait of the M2 macrophages [23]. As a possible anti-inflammatory effect, it has been proposed that it may compete for ECM binding with leukocytes and eventually inhibit leukocyte evasion [62]. However, the precise mechanism of Ym1 actions in macrophages is uncertain. It binds heparin and heparan sulfate, and due to its enzymatic activity, it might contribute to the lysis of glycosaminoglycans [59]. Heparan sulfate glycosaminoglycans are constituents of the macrophage glycocalyx and they have impact on the macrophage functions in disease [68]. For instance, diminished sulfation of heparan sulfate enhances chemokine expression in macrophages and increases foam cell—an M1-like macrophage type in atherosclerotic plaques—conversion [68]. The overexpression of heparanase (EC 3.2.1.166)—enzyme degrading heparan sulfate—is associated with increased expression of some M2 molecules, such as IL-10, chemokine (C-C) motif ligand 2 (CCL2), vascular endothelial growth factor (VEGF), and IL-6 in TAMs [69]. The amount of heparan sulfate is likely to affect Ym1 levels, since the lack of heparanase increases Ym1 accumulation in macrophages [61]. These findings suggest that Ym1 may play a role in the fine-tuning of macrophage heparan sulfate levels, which has impact on macrophage activation.

Ym1 may be involved in inflammatory response [60, 63] and also act as a danger-associated molecular signal [70]. It displays chemotactic activity for T lymphocytes and bone marrow cells and possibly for eosinophil granulocytes [60]; however, this effect may depend on the disease model [23]. It is also a substrate for the metalloproteinases MMP-2 and MMP-9, which may allow modulation of its chemotactic activity [71]. In dendritic cells Ym1 is needed for the initiation of Th2 immune response [72]. Ym1 also increases the number and the activity of IL-17 producing *γδ* T cells, which eventually leads to the recruitment of neutrophil granulocytes [70]. In helminth infection Ym1 thus limits parasite survival; however, it enhances tissue injury [70]. A recent study shows that Ym1 inhibits antiviral T-cell responses and is involved in the helminth-induced impairment of antiviral immunity [73]. It has been shown that the neutralization of Ym1 in mice coinfected with* Trichinella* and influenza virus enhances the virus-specific CD8^+^ T cell proliferation, and Ym1 inhibits activation and proliferation of CD8^+^ T cells* in vitro* [73].

M2a macrophages are often termed as wound-healing macrophages [6]; however, macrophages fail to fit into clear M1 or M2 categories in the course of wound healing [9]. The presence of Ym1 protein is shown in wound associated macrophages, without upregulation of its mRNA level [74]. A recent study shows that macrophages are prone to take up recombinant Ym1* in vitro* and Ym1 which is released by wound neutrophil granulocytes* in vivo* [74]. The ability of wound macrophages to take up Ym1 shows that Ym1 immunopositive macrophages are not necessarily M2 activated.

### 3.3. CD206 (C-Type Mannose Receptor 1) and CD163 (Hemoglobin-Haptoglobin Scavenger Receptor)

CD206, also termed as MRC1 (C-type mannose receptor 1), is an M2 macrophage marker in both the mouse and the human [10, 27]. CD206 is a 175-kDa type I transmembrane glycoprotein which binds and internalizes glycoproteins and collagen ligands. Several types of tissue resident macrophages express CD206 in the mouse and the human, such as cardiac resident macrophages, peritoneal macrophages, adipose tissue macrophages [75–78], placental macrophages (also known as Hofbauer cells) [79], and macrophages of the skin [80]. In tissue resident macrophages the CD206 expression can be maintained without the need of IL-4 receptors suggesting that the tissue environment promotes CD206 expression [80]. Its expression is amplified in intestinal helminth infections, by IL-4, granulocyte macrophage colony stimulating factor (GM-CSF), TGF-*β*, and other IL-4/IL-13 independent signal pathways [80]. CD206 has not yet fully understood immune functions; for instance, its lack increases random migration of macrophages and results in the upregulation of proinflammatory cytokine production during endotoxemic lung inflammation in the mouse [81]. The lack of CD206 also results in the elevated serum level of inflammatory proteins, suggesting that it has a role in the resolution of inflammation by clearing inflammatory molecules from the blood [82]. However, CD206 expressing macrophages have unfavorable profibrotic effects, since they promote fibroblast growth through TGF-*β* and chemokine (C-C) motif ligand 18 (CCL18) secretion [83]. They may also undergo a fibrocyte-like phenotype switch and produce collagen [84]. Nevertheless, this profibrotic role has some beneficial effects also, for example, in atherosclerotic plaques, where it may increase plaque stability thus avoiding plaque rupture [84]. Resident macrophages of the colon lamina propria constitutively express CD206 and secrete IL-10, possibly in response to stimuli of the gut microbiota [85]. Similarly, CD206 expressing human decidual macrophages produce IL-10 and CCL18 with a possible role in the maternal immunological tolerance of the fetus [79].

Some CD206 expressing tissue resident macrophages, such as mouse and human adipose tissue macrophages and placental macrophages, also express CD163, which is a haptoglobin-hemoglobin scavenger receptor [75, 76, 86, 87]. It is an M2 marker protein, principally due to its upregulated expression in response to IL-4 [10]. Its expression is amplified also by M-CSF, IL-6, IL-10, and glucocorticoids, while TNF-*α*, TGF-*β*, IFN-*γ*, and LPS reduce its expression [27, 88–90]. In human monocytes and resident macrophages CD163 has a high basal expression, amplified by IL-10 and glucocorticoids [89–91]. Surprisingly IL-4 represses or does not affect its expression [27, 90]. Macrophages coexpressing CD206 and CD163 are high IL-10, IL-1 receptor antagonist (IL-1ra), and CCL18 producers [79]. They also have high capacity of apoptotic cell uptake [92]. CD163 expression is increased in TAMs [93] and in peritonitis [83], and it is secreted into the blood in severe inflammation [94]. The expression of CD163 is not restricted to M2 macrophages; thus, it should not be used as a sole marker to identify the M2 activation [95].

### 3.4. Found in Inflammatory Zone 1 (FIZZ1)

Found in inflammatory zone 1 (FIZZ1), also known as hypoxia-induced mitogenic factor (HIMF) or resistin-like molecule *α* (RELM*α*), is a 9.4 kDa cysteine-rich secreted protein [23]. Its expression is upregulated by helminth infection, IL-4 and IL-13 via the STAT6 pathway, and suppressed by IFN-*γ* [23, 96]. In helminth infection FIZZ1 diminishes inflammation [97, 98]. However, FIZZ1 is abundant in the bronchoalveolar lavage fluid in allergic airway inflammation in the mouse, where it causes vascular inflammation, exhibits chemotactic and fibrogenic properties, induces myofibroblast differentiation, and recruits bone marrow-derived cells [23, 99–102]. In specific brain regions the microglia expresses FIZZ1 and its expression is highly upregulated by IL-4 [103]. However, its expression can be amplified in mice lacking the IL-4 receptor or STAT6, and its expression may be regulated by other signal transduction mechanisms, such as through the regulator of G protein signaling 10 [104].

### 3.5. Dendritic Cell Specific ICAM-3 Grabbing Nonintegrin (DC-SIGN) or CD209

Macrophage expression of dendritic cell specific ICAM-3 grabbing nonintegrin (DC-SIGN), also known as CD209, is increased by IL-4 [105]. Inflammatory signals, including IFN-*γ* and TGF-*β*, diminish the effect of IL-4 on its expression [105]. M-CSF and IL-10 amplify its expression [88]. It is a marker of dendritic cells; however, certain tissue resident macrophages express it, such as colorectal mucosal macrophages, placental macrophages, alveolar macrophages, and adipose tissue macrophages in the mouse [88, 106–109]. In the presence of M-CSF, combined IL-4 and IL-13 treatment induces CD209 expression in human microglia cultured* in vitro* [110]. Its functions have been studied mainly in dendritic cells, where it plays a broad range of immune roles, such as migration, T cell activation, antigen internalization, and binding of various pathogens and tumor cells [111].

### 3.6. Galactose-Type C-Type Lectin (MGL-1) and Dectin-1

The macrophage galactose-type C-type lectin (MGL) gene family members recognize glycan structures in a Ca^2+^-dependent manner through a carbohydrate recognition domain [112]. They are implicated in the uptake of glycoproteins, immune cell interactions, and pathogen recognition [113]. In the mouse MGL1 and MGL2 are expressed in peritoneal macrophages elicited during parasite infection and in alveolar macrophages in allergic asthma. IL-4 and IL-13 upregulate both MGL1 and MGL2 expressions [113]. However, MGL1 is predominant in mouse macrophages, where its function may be the antigen recognition of helminth parasites, and the inhibition of TNF-*α* and IL-12 gene transcription [113]. In mouse adipose tissue macrophages, high fat diet feeding increases MGL-1 expression, congruent with the mixed expression of M1 and M2 markers [87]. The human homologue of MGL1 is MGL, which recognizes antigens and increases IL-10 and TNF-*α* expression [113]. It is expressed by TAMs also [114].

Dectin-1 (CLEC7A) is a lectin-like innate immune receptor, which binds beta-glucans [115]. The fungal cell wall is rich in beta-glucans; thus, it has a key role in the recognition and phagocytosis of pathogenic fungi by macrophages [116]. Tumor cell surfaces can express N-glycans, which are also recognised by dectin-1, allowing the uptake of tumor cells [117]. Absence of dectin-1 impairs the phagocytic and fungicidal abilities of macrophages and alters nitric oxide and cytokine production. Increasing dectin-1 expression improves antifungal defense [118]. It is suggested that M2a macrophages have high dectin-1 expression, while M2b macrophages express low levels of dectin-1 [116]. However, contrary to expectations, the lack of dectin-1 amplifies the expression of other M2 markers such as Ym1, arginase-1, and FIZZ1 [118]. Of note, dectin-1 is involved in the M1 macrophage activation as well and increases pathogen killing [118]. In the mouse, various resident macrophage types express dectin-1: alveolar macrophages, Kupffer cells, intestinal macrophages, and splenic macrophages, with a possible role in pathogen recognition [119].

### 3.7. Neurotransmitters, Hormones, and Growth Factors

The M2 macrophages are sources of neurotransmitters and hormones, such as catecholamines [120], substance P [121], adiponectin [122], and growth factors [123]. Therefore, they are parts of the diffuse or tissue neuroendocrine system.

Catecholamine synthesis is a recently recognized trait of M2 macrophages [120, 124]. Catecholamines produced by adipose tissue macrophages have metabolic impact, by promoting brown adipose tissue differentiation and adaptive thermogenesis in the mouse [120, 124]. The expression of arginase-1 is increased, while NO synthesis is inhibited by catecholamines, making it likely that they promote M2 activation [125, 126]. M2 macrophages also produce insulin-like growth factor-1 (IGF-1) [106, 127, 128], which can help tissue regeneration [127] and have a role in sustaining M2 activation [129]. Adiponectin gene expression has been shown in mouse adipose tissue macrophages, and its transcript level is diminished when M2 activation is compromised [122]. Expression of adiponectin receptors correlates with the activation state, and adiponectin has activation state dependent effects in macrophages [130]. Adiponectin can shift macrophages into M2 activation [131], and in the M2 activation state, adiponectin increases IL-10 synthesis, while in M1 activation it has an overt inflammatory effect, by promoting the expression of TNF-*α*, IL-6, and IL-12 [130].

Substance P, a tachykinin neuropeptide, is released from inflammatory cells including dendritic cells and macrophages, and nerve endings at the site of inflammation in the respiratory, gastrointestinal, and musculoskeletal systems [121]. LPS and subsequent nuclear factor kappa beta (NF-*κ*B) activation induce macrophage substance P expression, while TGF-*β* has the opposite effect [121, 132]. Substance P synthesis is thus associated with M1 activation of the macrophages. However, recent data suggest that substance P may also be associated with the M2 activation [133–135]. Constitutive substance P expression has been reported in mouse intestinal macrophages, and it is induced by helminth infection in splenic macrophages [121]. Interestingly, substance P is able to shift macrophages to an M2 activation state in rat spinal cord injury and induce CD163 expression in human macrophages [133, 134]. Vasoactive intestinal peptide (VIP) is synthesized by macrophages and it can increase the secretion of IL-10 by macrophages [136]. However, its expression is repressed by IL-4 [137]. Neuropeptide Y is produced by adipose tissue macrophages, and macrophages lacking its expression have increased inflammatory cytokine production [138, 139].

CD206 positive macrophages produce hepatocyte growth factor in injured muscle, which may help muscle fiber regeneration [123]. Other angiogenic growth factors are also produced by M2 macrophages, such as basic fibroblast growth factor, IGF-1, chemokine (C-C) motif ligand 2, placental growth factor, and vascular growth factor-A (VEGF-A) [127]. In normal tissue development the M2 macrophages have a key role in the angiogenesis of the developing tissues [2], and a recent study suggests that specific M2 macrophage subsets (such as M2a and M2c) may act through distinct signal pathways to promote angiogenesis [127]. In wound healing the synthesis of angiogenic factors supports the blood supply of the regenerating tissue. However, in tumor tissue the M2 macrophages support the vascularization and the survival of the tumor tissue [140].

### 3.8. Lipid Metabolites

Macrophages synthesize lipid derivatives, such as omega-3 fatty acids, lipoxins, and palmitoleic acid, which have anti-inflammatory effects and may also be involved in the antiviral response of macrophages [141–143]. They act on other immune cells through elaborating lipid mediators or may have autocrine effects on macrophages [142, 144]. For instance, lipoxin A4 stimulates apoptotic cell uptake by neutrophil granulocytes and reduces inflammatory cytokine expression possibly through impeding NF-*κ*B signaling [145]. A lipoxin A4 derivative protects macrophages from LPS-induced apoptosis and reverses the effects of LPS on macrophage potassium currents [146]. Altogether, these effects of lipoxins may promote the resolution of inflammation and counteract M1 activation [147]. Similarly, endogenously produced omega 3 fatty acids inhibit the NF-*κ*B dependent inflammatory response in macrophages [142]. The synthesis of anti-inflammatory lipid metabolites is an interesting trait of macrophages and may have therapeutic impact by resolving adipose tissue inflammation and impeding insulin resistance by promoting M2 activation [78, 147, 148]. However, to date it is still a largely unexplored area of research in the macrophage field. Anti-inflammatory lipids produced by macrophages are not listed as M2 markers in the literature. However, in human monocytes IL-13 upregulates 15-lipoxygenase, the enzyme producing lipoxin A4 [149]. Also, the M1 and the M2 macrophages have characteristic lipid mediator signatures [150]; moreover, the distinct macrophage types have differences in the transcription of genes involved in the synthesis of lipid mediators [151]. Thus, it is likely that M2 activation is associated with increased production of anti-inflammatory lipid derivatives.

### 3.9. Other Molecules Associated with M2 Macrophages

The cytokine and chemokine profile of macrophages can define their activation state [10]. M2 macrophages secrete anti-inflammatory cytokines IL-10 and IL-1ra, which may distinguish them from M1 macrophages. However, cytokines associated with M1 activation may be produced by M2 macrophages also, such as IL-6, TNF-*α*, and IL-12 [8, 10]. There are many other molecules, which have been proposed as markers of M2 activation, such as chemokine C-C motif ligand 17 (CCL17), CD200R, or CD23 [152, 153]. M2 activation of lung macrophages is associated with CCL17 expression in asthma [153]. CD200R is expressed by mouse peritoneal macrophages in helminth infections and may be expressed by the microglia; however, its function in macrophages is to be defined [152]. CD23 is a low affinity IgE receptor, with the ability to regulate cytokine expression in macrophages. Its expression is amplified by IL-4 in human monocytes and is expressed by alveolar macrophages with a role in allergic response [154, 155]. TAMs produce chemotactic factors which promote tumor cell motility [156]. Molecules associated with phagocytosis of apoptotic cells also hallmark M2 macrophages, such as galectin-3 [157], Mer tyrosine kinase (MERTK), Axl receptor tyrosine kinase, and growth arrest-specific 6 (Gas-6) [92, 158], signaling lymphocyte-activation molecule (SLAM) [92, 158]. The uptake and consequent digestion of the apoptotic cells may produce lipid and retinoid derivatives which may activate ligand sensitive transcription factors, such as peroxisome proliferator activator receptors (PPARs), liver X receptors (LXRs), or retinoid X receptors (RXRs) [158–160]. These transcription factors promote the gene transcription of M2 associated genes, aid further apoptotic cell uptake, and can repress genes of M1 activation [158, 161, 162]. Recently, it has been shown that the multifunctional enzyme transglutaminase 2 (TGM2) is associated with apoptotic cell uptake [158, 163] and may be a marker of M2 macrophages [4]. Antigen presentation can also be a task of M2 macrophages, depending on the context of the M2 activation. Thus, antigen presenting M2 macrophages also express major histocompatibility complex-II (MHC-II) [10].

## 4. Pattern of M2 Markers in Embryonic and Postnatal Development

Constitutive expression of M2 markers by tissue resident macrophages has a specific pattern in embryonic and postnatal development. For instance, embryonic development of the microglia and the brain perivascular macrophages is associated with the expression of CD200R [164]. Microglia arginase-1 expression has an age-dependent pattern in the mouse, with a peak in postnatal day 3 [165]. In the developing rat liver the number of CD163 expressing macrophages inclines after birth and is maintained in adulthood [166]. In the porcine embryo, the liver, the lungs, and the spleen contain CD163 positive macrophages [167]. CD163 is also expressed by pluripotent, fibrocyte-like macrophages of the umbilical cord [168]. Ym1 is expressed in early hematopoietic progenitors in the mouse embryo, and the lung is seeded by Ym1 expressing macrophages from embryonic day 18.5 [64]. Macrophages of the mouse lung adopt an M2 phenotype at the period of lung alveolarization at postnatal days 14–21. In this period the lung macrophages have upregulated expression of arginase-1, CD206, and CCL17 [128]. Despite the expression of these M2 markers in embryonic macrophages, there is no consensus whether the M1/M2 model of macrophage activation should be extended to the embryonic macrophages. Aging diminishes the responsiveness of macrophages to activation stimuli, and IL-4 exposure results in a blunted arginase-1, Ym1, and FIZZ1 expression in adherent splenocytes from aged mice compared with younger animals [169].

## 5. Development of M2 Macrophages: Phenotype Plasticity or Lineage Determination

Macrophages with M2 characteristics have high functional heterogeneity and occur in distinct organs under steady-state conditions. However, it is still not well understood whether this heterogeneity is a result of their reversible adoption of M2 activation in response to the tissue environment or due to irreversible differentiation programs.

The tissue environment is a source of signals with strong potential to shape macrophage activation [80]. Conditioned medium of adipocytes, the presence of cell-cell contacts (e.g., microglia cell-cell contacts [165], Kupffer cell contacts with hepatic stellate cells [170]) determine the expression level of M2 markers. Also, there is a growing number of tissue-derived endocrine signals with the potential to promote M2 activation, independently of the IL-4 receptor/STAT6 pathway [171, 172]. The proximity of nerve terminals to tissue resident macrophages supports the idea that the neuroendocrine system can control macrophage activation [173]. Hormones and neurotransmitters such as catecholamines [125], acetylcholine [174], glucocorticoids, adrenocorticotropic hormone, dihydrotestosterone [175], substance P [133, 135], VIP [136, 176], pituitary adenylate cyclase activating protein (PACAP) [136], neuropeptide FF [177], neuropeptide Y [40], adiponectin [13, 178], leptin [179], and adenosine [26] promote M2-like activation with impeding acquisition of M1 activation. Apoptotic cells, microparticles released from platelets [180], and pyrophosphate [181] are also elicitors of M2 activation in the tissues. The normal flora also has impact on M2 activation of tissue resident macrophages [182]. This possibility is supported by studies in germ free mice; however, the impact of microbiota may be different in distinct tissues. For instance, in germ free mice the M2 activation is increased in wound healing [183]; however, M2-like features of colon resident macrophages and the accumulation of adipose tissue macrophages are blunted [184]. There are also examples showing that tissue signals may impede M2 activation. For instance, Met-enkephalin inhibits arginase-1 and CD206 [41], and angiotensin diminishes Ym1 expression [185]. Tissues can contain mixed macrophage populations, and it is also likely that signals elaborated by M2 macrophages affect the net activation state of the tissue macrophage pool. For instance, M2 activated liver Kupffer cells increase the apoptosis of M1 activated Kupffer cells [186].

Until very recently the tissue resident macrophages were considered as descendants of bone marrow hematopoietic stem cells. However, it is shown that the resident tissue macrophages have heterogeneous progeny [187, 188]. The microglia develops from stem cells derived from the embryonic yolk sac; the heart contains a mixed resident macrophage population deriving from both yolk sac progenitors and bone marrow stem cells, while the skin and the gut resident macrophages are replenished from the bone marrow [187, 189–191]. These findings show that the differentiation of the tissue resident macrophages is diversified before birth, giving rise to separate cell lineages. Within this novel paradigm one should consider that the M2-like macrophage pools of the distinct tissues may be segregated by distinct differentiation programs. This would change the canonical view that M2 activation is shaped mainly by tissue environment and immune signals. However, to date there is no specific study available which would test this possibility. The M2 activation is controlled by transcription factors, such as IRF4, PPARs, RXRs, and LXRs, which determine the lineage commitment of immune cells [161, 192, 193]. In addition, the most recently discovered impact of microRNAs on myeloid cell development and M2 activation [194] also suggests that complex differentiation programs may cause the heterogeneity of M2 macrophages.

## 6. Evolutionary Roots of the M2 Macrophage Phenotype

We have only a few data sets on M2 macrophages in mammals outside of rodents and humans [167]. For instance, equine myeloid cells express CD163, CD206 [195], and CD23 [196], and porcine macrophages express CD163 [167]. Macrophages of the rainbow trout* Oncorhynchus mykiss* produce lipoxins [197]; however, we lack comparative studies showing that vertebrates other than mammals have M2-like macrophages. Looking at a wider horizon, however, we can recognize some M2-like traits of the invertebrate immune cells. Invertebrates have phagocytosing immune cells, termed with various names in the literature, such as hemocytes, coelomocytes, amoebocytes, and phagocytes. These immune cells are present in the hemolymph and can also be settled in the tissues [198]. They have primary functions in the innate immunity through phagocytosis of pathogens, secretion of pathogen-binding and pathogen-killing substances, antigen processing, and antigen presentation [198]. Like the M2 macrophages, they can be activated by parasites and fungal cells, and they facilitate wound healing and have some extent of impact on the metabolic performance of the tissues [198]. In wound healing, the hemocytes undergo a phenotype switch and adopt a collagen-producing, fibroblast-like phenotype [199]. Similar transition to collagen producing cells is known for monocytes in fibrosis [200].

Beyond these general functional similarities to the mammalian tissue resident and M2 macrophages, orthologs of some M2 marker genes are also known in invertebrate phagocytes. For instance, hemocytes of the* Pacifastacus leniusculus* crayfish contain mannose receptor protein, which is secreted in response to infection [201]. The oyster* Crassostrea gigas* hemocytes express chitinase-like proteins, which may support tissue growth and remodeling [202]. A secreted 47-kDa glycoprotein, DS47, isolated from* Drosophila melanogaster* Schneider line-2 cells—a line exhibiting macrophage-like properties—shares homology with the Ym1 [203]. Chitinases of the invertebrate phagocytes may have a role in pathogen defense [202]. However, invertebrate phagocytes are also capable of chitin synthesis [204], and to date it is uncertain whether the chitinase activity is associated with the control of their endogenous chitin storage. Invertebrate phagocytes metabolize L-arginine, and arginase expression has been shown in the shrimp* Penaeus monodon* hemocytes [205]. In invertebrate tissue, expression of arginase is induced by parasite antigens and TGF-*β* signals, and this is associated with collagen synthesis [33].

All these traits of the invertebrate hemocytes suggest that they can adopt a phenotype combating parasites and fungal cells and also a matrix producing and tissue healing phenotype. This makes them similar to the M2 macrophages. Possible signals, which may be responsible for such M2-homolog hemocyte activation, are platelet-derived growth factor/vascular endothelial growth factor (PDGF/VEGF) family proteins and TGF*β*. PDGF/VEGF proteins have been implicated in cell proliferation, cell differentiation, and cell migration. In* Drosophila melanogaster* the VEGF/PDGF ligands synthesized by the developing Malpighian tubules attract hemocytes [206]. The settled hemocytes secrete matrix components of the basement membrane [206]. A gene encoding a PDGF/VEGF related factor has been cloned in the crab* Eriocheir sinensis* [207]. It is expressed by hemocytes, and it provokes the release of noradrenaline and dopamine [207]. This trait resembles the catecholamine release of adipose tissue macrophages in the mouse [120]. In the snail* Limax maximus* the hemocytes contain PDGF and TGF-*β* immunoreactive material [199]. Exogenous administration of PDGF and TGF-*β* stimulates the tissue healing process [199].

Based on these findings we can draw the conclusion that invertebrate hemocytes have functions in extracellular matrix synthesis and wound healing and have endocrine functions beyond their role in pathogen defense. This means that invertebrate hemocytes possess characteristics of the mammalian M2 macrophages. However, viral, fungal, and bacterial infections elicit overlapping transcriptional changes in the invertebrate hemocytes [198] which suggests that an M1-M2 separation is lacking in the invertebrate hemocytes.

## 7. Summary and Perspectives

The findings reviewed here show that the M2 terminology covers a functionally diverse group of macrophages, rather than a uniform activation state. Contrary to the prevailing model which depicts M1 and M2 activation as “black and white,” strikingly different and functionally distinct states of macrophages [10], the recent progress gives colors and tones to this image. The M2 macrophages undertake host defense and wound healing/tissue remodeling tasks, with additional contributions to the metabolic performance and the endocrine signaling of the tissues. This functional heterogeneity is associated with a similar heterogeneity of the expression of the M2 markers discussed here. The analysis of global gene transcriptional changes, lipid composition, and metabolomic signature will further refine the image of the M2 macrophage functions. It is easy to predict that an alternative classification of the macrophage activation states will substitute the generalized M2 terminology with more accurately defined divisions of the functionally distinct macrophages.
